# Virotherapy for Spinal and Spinal Cord Tumors: Current Evidence and Future Perspectives

**DOI:** 10.3390/microorganisms14092110

**Published:** 2026-09-21

**Authors:** Koji Uotani, Tomohiro Fujiwara, Ryo Takatori, Kazutaka Yamashita, Kenzaburo Matsumoto, Yoshiaki Oda, Kensuke Shinohara, Hiroshi Tazawa, Toshiyoshi Fujiwara, Toshifumi Ozaki

**Affiliations:** 1 Department of Orthopaedic Surgery, Okayama University Graduate School of Medicine, Dentistry and Pharmaceutical Sciences, Okayama 700-8558, Japan; 2 Department of Gastroenterological Surgery, Okayama University Graduate School of Medicine, Dentistry and Pharmaceutical Sciences, Okayama 700-8558, Japan

**Keywords:** oncolytic virotherapy, spinal cord tumor, spinal metastasis, oncolytic adenovirus, OBP-301, hTERT, telomerase, glioma, blood–spinal cord barrier, cancer immunotherapy

## Abstract

Tumors of the spine and spinal cord, including primary and metastatic vertebral tumors, intramedullary gliomas, and refractory intradural lesions, are challenging to treat because curative local therapy is limited by the eloquent, nonregenerating neural tissue surrounding them. Oncolytic virotherapy may offer a rational alternative by combining tumor-selective oncolysis with the induction of systemic antitumor immunity, while sparing normal neural cells. This review summarizes the current field of oncolytic viruses, including adenovirus, herpes simplex virus, reovirus, and others, in the context of spinal and spinal cord tumors. Clinical data in the spine remain scarce; however, the rationale is based on histological evidence from sarcomas and other tumors, extensive glioma experience, including diffuse intrinsic pontine glioma, and preclinical activity in nerve sheath and meningioma models. We discuss the telomerase-specific oncolytic adenovirus OBP-301 and its derivatives, whose *hTERT*-driven replication provides histology-agnostic tumor selectivity, while sparing the telomerase-silent spinal cord. This telomerase dependence, however, limits activity against tumors that maintain telomeres through the alternative lengthening of telomeres (ALT) pathway, such as many osteosarcomas and some spinal cord astrocytomas. We also discuss delivery and safety within the confined spinal canal, combination strategies, and future directions, such as extracellular vesicle-mediated delivery and biomarker-guided patient selection. Although clinical translation to the spine will require dedicated preclinical and early-phase studies, virotherapy represents a promising, mechanistically grounded modality for these therapeutically challenging tumors.

## 1. Introduction

**Figure 1:**
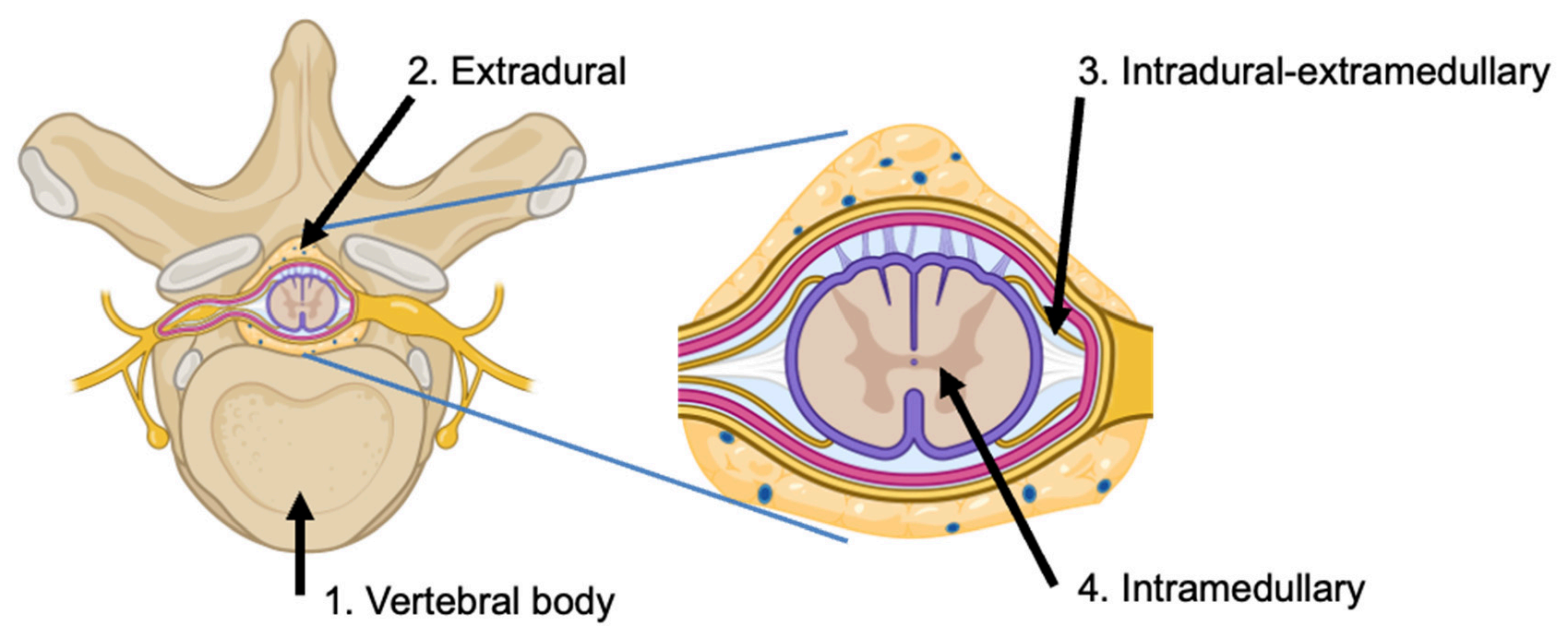
Anatomical compartments of spinal and spinal cord tumors. The tumors are grouped by compartment: vertebral body (primary bone tumors and metastases), extradural space (metastatic epidural spinal cord compression), intradural–extramedullary compartment (meningioma and schwannoma, with malignant or recurrent lesions as a selected niche), and intramedullary compartment (astrocytoma and ependymoma). The filled circles mark the principal focus of this review.

Tumors that affect the spine present two distinct anatomical problems for surgeons: neoplasms of the vertebral (bony) column and tumors of the spinal cord and its coverings [1]. Vertebral column tumors include rare primary bone tumors, such as osteosarcoma, Ewing sarcoma, chordoma, chondrosarcoma, and giant cell tumor of bone, as well as the far more common metastatic spinal tumors, of which approximately 18,000 new cases are diagnosed annually in North America [2,3]. Tumors of the spinal cord are classified as intramedullary, intradural–extramedullary, or extradural, based on compartment (Figure 1). Intramedullary spinal cord tumors (IMSCTs), which are predominantly ependymomas in adults and astrocytomas in children, are rare and account for just 2–4% of central nervous system (CNS) neoplasms [4]. Among these compartments, the tumor arises within or immediately adjacent to the eloquent, nonexpandable neural tissue, a feature that prevents effective treatment.

These anatomical constraints limit each pillar of conventional therapy. For well-demarcated lesions, such as spinal ependymomas, gross-total resection is associated with better survival and durable local control [4,5]. In contrast, infiltrative astrocytomas lack a surgical plane, and aggressive resection risks irreversible motor and sensory deficits. High-grade histology confers an approximately 14-fold higher risk of death compared with low-grade disease [4]. Primary vertebral bone tumors often require *en bloc* resection to achieve negative margins; however, this is a technically demanding procedure with substantial morbidity, and outcomes remain poor for high-grade and recurrent disease [6]. Radiotherapy is limited by the low radiation tolerance of the spinal cord. Conventional systemic chemotherapy shows modest efficacy against most of these tumors [5]. For spinal metastases, treatment is largely palliative, whereas the confined spinal canal magnifies the neurological consequences of progression [3]. Collectively, these limitations define an unmet need for locally active, tumor-selective therapies that eradicate tumors while sparing adjacent neural tissue.

Oncolytic virotherapy represents a rational strategy to meet this need. Oncolytic viruses (OVs) selectively replicate in and lyse tumor cells, while sparing normal tissue. The resulting immunogenic cell death converts an immunologically “cold” tumor microenvironment into an inflamed, immune-reactive state that promotes systemic antitumor immunity [7,8,9,10]. The viability of this approach has been established. For example, talimogene laherparepvec, a granulocyte–macrophage colony-stimulating factor–armed herpes simplex virus type 1 (HSV-1), was approved for the treatment of advanced melanoma in 2015 [11]. With respect to neuro-oncology, the conditionally replicating HSV-1 teserpaturev (G47Δ) was approved in Japan in 2021 as the first oncolytic virus for the treatment of a primary brain tumor, malignant glioma [12]. Similarly, the oncolytic adenovirus DNX-2401 produced encouraging survival signals in recurrent glioblastoma, including in combination with immune-checkpoint blockade [13]. Taken together, these achievements indicate the feasibility of delivering virotherapy within the CNS.

Of the OV platforms, oncolytic adenoviruses offer favorable properties for treating spinal and CNS tumors, including a large transgene capacity, non-integrating replication, and a well-characterized safety profile [14]. We developed a telomerase-specific, replication-competent oncolytic adenovirus, OBP-301 (suratadenoturev), in which the human telomerase reverse transcriptase (*hTERT*) promoter drives the viral *E1A* and *E1B* genes [15]. Because telomerase is reactivated in most human malignancies, but remains largely silent in normal somatic cells, OBP-301 preferentially replicates in tumor cells with diverse histologies. Moreover, its safety and antitumor activity have been confirmed in a first-in-human phase I trial [16]. A green fluorescent protein–expressing variant, OBP-401, maintains this tumor selectivity while enabling visualization of viral replication [17]. We also extended the fluorescence-based assessment of viral susceptibility to bone and soft-tissue sarcomas [18]. This work establishes the *hTERT*-driven adenoviral platform as a versatile, tumor-selective agent relevant to the histologies encountered in the spine.

Despite this progress, the application of virotherapy to spinal and spinal cord tumors remains largely unexplored, and no comprehensive synthesis of this field exists. This gap is notable because OVs have already shown activity against the histological types that give rise to primary vertebral tumors, including osteosarcoma, Ewing sarcoma, and chordoma, yet virotherapy directed specifically at spinal or paraspinal disease has not been widely reported [19,20,21]. These tumors exhibit features that are well matched to OVs. For example, they are frequently localized and accessible for intratumoral injection; they demand tumor-selective cytotoxicity to preserve neural function, and most express molecular targets, such as *hTERT*, that OV designs can exploit. In this review, we summarize the current evidence for oncolytic virotherapy, spanning adenovirus, herpes simplex virus, reovirus, and other platforms, across the spectrum of spinal and spinal cord tumors, and encompassing primary and metastatic vertebral tumors as well as intramedullary, intradural–extramedullary, and extradural lesions. We also discuss the considerations of viral delivery and safety within the confined spinal canal, including the blood–spinal cord barrier, and outline the challenges and future directions for translating this approach into clinical practice.

## 2. Clinical Landscape and Unmet Needs of Spinal and Spinal Cord Tumors

### 2.1. Primary Vertebral Bone Tumors: Clinical Overview

Primary malignant tumors of the vertebral column are rare. The most frequently encountered histologies are chordoma, chondrosarcoma, osteosarcoma, and Ewing sarcoma [6]. These tumors often present with nonspecific back pain; therefore, their diagnosis is frequently delayed until neurological deficit or spinal instability develops [6]. For most primary malignant vertebral tumors, *en bloc* resection with tumor-free margins offers the best chance of local control and cure. Surgical planning is guided by the Enneking and Weinstein–Boriani–Biagini (WBB) staging systems [22,23]; however, *en bloc* resection in the spine is challenging and carries a high complication rate because of the surrounding neural and vascular structures. In addition, negative margins are often unattainable in anatomically constrained or recurrent disease [24]. Adjuvant options are limited. For example, osteosarcoma and Ewing sarcoma require systemic chemotherapy, chordoma and chondrosarcoma are relatively radioresistant and chemoresistant, and outcomes for high-grade or recurrent tumors are poor [6]. These features create a strong rationale for tumor-selective local therapies.

### 2.2. Metastatic Spinal Tumors: Clinical Overview

Metastatic disease is the most common tumor of the spine. The incidence continues to increase as systemic therapy prolongs survival in patients with advanced cancer [3]. The vertebral column is the most common site of skeletal metastasis, and epidural extension can produce metastatic epidural spinal cord compression, which is a neurological emergency [25]. Contemporary management is multidisciplinary and is organized by decision frameworks, such as NOMS (neurologic, oncologic, mechanical, systemic) and the Spinal Instability Neoplastic Score (SINS) [26,27]. The therapeutic paradigm has shifted from aggressive cytoreduction toward “separation surgery” (i.e., circumferential decompression of the cord), followed by stereotactic body radiotherapy (SBRT), which can achieve durable local control with lower morbidity [25]. Nevertheless, treatment is primarily palliative, and the overall prognosis remains guarded—often measured in months rather than years, and shorter still with epidural spread—though it varies widely with the primary tumor, disease burden, and performance status. A locally active therapy that also engages systemic antitumor immunity is needed to address this gap.

### 2.3. Intramedullary Spinal Cord Tumors

IMSCTs are rare. They comprise 2–4% of all CNS neoplasms. Ependymomas predominate in adults and astrocytomas in children [4]. For circumscribed ependymomas, gross-total resection results in favorable long-term local control and survival [4,5]. In contrast, astrocytomas are infiltrative and lack a surgical dissection plane. Therefore, aggressive resection risks irreversible neurological injury, and high-grade histology carries an approximately 14-fold higher risk of death compared with low-grade disease [4]. The low radiation tolerance of the spinal cord hinders adjuvant radiotherapy, and conventional chemotherapy has limited efficacy [5]. Consequently, high-grade IMSCTs have a poor prognosis and represent a compelling target for tumor-selective virotherapy.

### 2.4. Intradural–Extramedullary and Extradural Tumors

Intradural–extramedullary tumors, which are predominantly meningiomas and schwannomas, are usually benign and are effectively managed by resection; thus, they are not candidates for virotherapy [28]. An important exception is recurrent, atypical, or malignant meningioma that adheres densely to the spinal cord, in which gross-total resection is precluded and repeat surgery or radiotherapy carries an increased neurological risk. Notably, telomerase reactivation and TERT promoter mutations are prevalent in aggressive, recurrence-prone meningiomas independent of WHO grade [29,30]. This provides a rationale for *hTERT*-driven virotherapy in this specific refractory subgroup, although direct evidence in meningioma is lacking. They are also relevant because their location illustrates the surgical anatomy and the intradural and intrathecal delivery corridors that any spinal virotherapy must navigate [28]. Extradural tumors are dominated by the metastatic and primary bony lesions described above along with rare aggressive entities. Therefore, this review focuses on the malignant intramedullary and vertebral tumors and on selected refractory intradural lesions, for which the unmet need is greatest.

### 2.5. Limitations of Current Treatment and Rationale for Virotherapy

Across every compartment, the same constraint recurs. Curative-intent local therapy is limited by the immediate proximity of functionally important, poorly regenerating neural tissue. Surgery is bound by the need to preserve neurological function, radiotherapy by low radiation tolerance of the spinal cord, and systemic therapy by the modest efficacy of chemotherapy against most histologies [4,5,6,25]. An ideal therapy would combine local tumor selectivity with the capacity to elicit systemic antitumor immunity, while sparing the surrounding neural structures. Oncolytic virotherapy embodies these properties, and the sections that follow discuss its mechanistic basis, principal viral platforms, and evidence for its application to spinal and spinal cord tumors.

## 3. Principles of Oncolytic Virotherapy

### 3.1. Selective Viral Replication and Direct Oncolysis

The therapeutic selectivity of OVs depends on molecular differences between the tumor and normal cells. Malignant cells frequently exhibit defective antiviral interferon signaling, a dysregulated cell cycle, and activated oncogenic pathways, all of which promote viral replication [7,8]. Two complementary strategies exploit these differences. Some viruses, such as reovirus, Newcastle disease virus, and vesicular stomatitis virus, contain an inherent preference for cells with defective interferon responses or activated RAS signaling [7]. Others are genetically engineered for tumor selectivity, either by deleting viral genes that are dispensable in tumor cells but required for replication in normal cells, for example, the *E1B* or *E1A*-CR2 deletions in adenovirus and the ICP34.5 deletion in herpes simplex virus, or by inserting essential viral genes under the control of tumor-specific promoters [13,15]. Regardless of the mechanism, productive replication amplifies the input dose within the tumor, which spreads to neighboring malignant cells, and culminates in direct oncolysis [7,8].

### 3.2. Immunogenic Cell Death and Antitumor Immunity

Oncolysis is not merely a cytotoxic event, but a potent immunological stimulus. Lysis releases damage-associated molecular patterns, pathogen-associated molecular patterns from the virus, and tumor-associated antigens, which activate dendritic cells and prime tumor-specific cytotoxic T lymphocytes [9,10]. This process, known as immunogenic cell death, converts an immunologically “cold,” T-cell–excluded tumor into an inflamed, “hot” microenvironment that generates systemic, abscopal responses against distant disease [9,10]. The immune effect may be amplified by arming the virus with immunostimulatory transgenes, as exemplified by the granulocyte–macrophage colony-stimulating factor encoded in talimogene laherparepvec [11]. This dual action, local oncolysis coupled with systemic immune activation, provides a rationale for combining OVs with immune-checkpoint inhibitors.

### 3.3. Determinants of Tumor Selectivity and Cell Entry

Two factors govern whether a particular tumor will respond: cell-surface receptor availability and the transcriptional environment. Cell entry relies on specific receptors: the coxsackievirus–adenovirus receptor (CAR) and integrins for adenovirus serotype 5, nectin-1 and HVEM for herpes simplex virus, and CD46 for measles virus [31,32]. CAR expression varies considerably among tumors and influences susceptibility to adenoviral infection. Notably, CAR is frequently downregulated in poorly differentiated, high-grade tumors, which may confer intrinsic resistance to adenovirus serotype 5–based agents [31,33]. Fiber-knob modifications, such as the RGD motif or Ad5/35 chimerism, enable CAR-independent entry and have been designed to overcome this heterogeneity [13]. In contrast, herpes simplex virus enters cells through the widely expressed receptors, nectin-1 and HVEM; thus, oncolytic HSV-1 primarily achieves tumor selectivity through engineered attenuation, such as deletion of the neurovirulence gene ICP34.5, rather than through receptor restriction [32]. Nevertheless, nectin-1, the principal entry receptor, is differentially expressed among tumors and predicts susceptibility to oncolytic HSV, including in nerve sheath tumors relevant to the spine. Conversely, its broad expression in neural tissue underlies the neurotropism that must be attenuated before intraspinal application [32,34].

**Figure 2:**
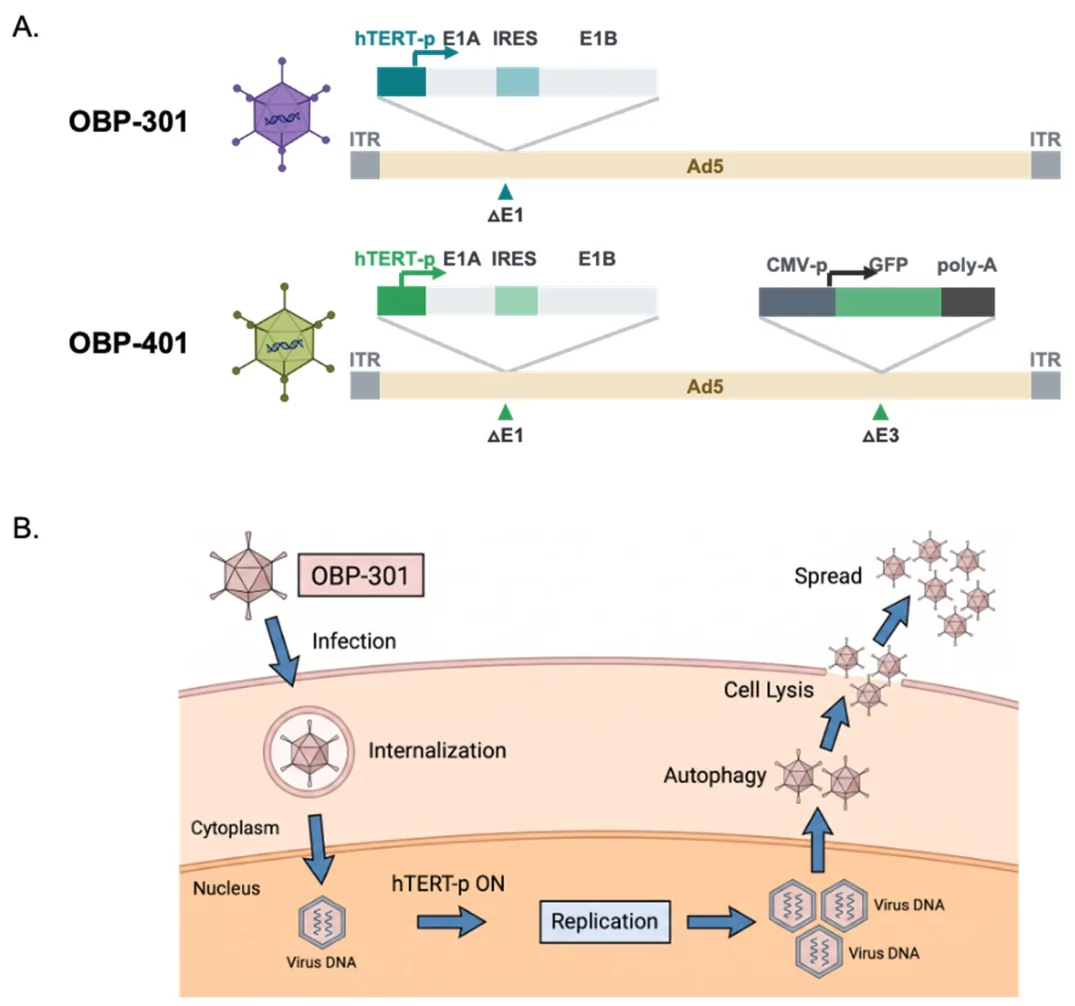
Telomerase-specific oncolytic adenovirus platform (OBP series). (**A**) In OBP-301, the human telomerase reverse transcriptase (*hTERT*) promoter drives the adenoviral *E1A* and *E1B* genes. OBP-401 also expresses green fluorescent protein (GFP). (**B**) Because *hTERT* is active in telomerase-positive tumor cells, but silent in post-mitotic neurons and glia, replication and oncolysis are confined to tumor cells, while the spinal cord is spared. ALT-positive (telomerase-negative) tumors exhibit decreased susceptibility.

Transcriptional targeting adds a second layer of selectivity. The *hTERT* promoter is particularly attractive because telomerase is reactivated in the majority of human malignancies; however, it is silent in most normal somatic cells, including the neurons and glia of the spinal cord [15,16]. Telomerase reactivation is not universal. Approximately 10–15% of cancers are telomerase-negative and maintain their telomeres through alternative lengthening of telomeres (ALT), which is a mechanism common in tumors of mesenchymal and glial origin. ALT has been reported in 25–60% of sarcomas, and in 47–80% of osteosarcomas, as well as in a substantial subset of astrocytomas [35,36]. Because *hTERT*-driven viruses depend on telomerase activity, ALT-positive tumors may be less susceptible, which is relevant to primary vertebral bone tumors and spinal cord astrocytomas. Therefore, inserting viral replication genes under *hTERT* control confines replication to telomerase-positive tumor cells, which is the design principle of OBP-301 and OBP-401. In adenoviral systems, oncolysis proceeds in part through autophagic cell death [37,38] (Figure 2). These findings indicate that receptor status and telomere-maintenance mechanisms vary with histology and grade; therefore, biomarker-guided assessment of CAR and telomerase/*hTERT* status is important to identify the spinal tumors that likely respond (Section 8). In the spine, this combination of receptor-dependent entry and telomerase-restricted replication offers a means to achieve tumor-selective cytotoxicity, while sparing adjacent neural tissue.

## 4. Oncolytic Virus Platforms: A Concise Primer

A range of viral platforms has been engineered or selected for oncolytic use. Several have a clinical track record already in the CNS, which is informative for spinal application. In this section, we summarize the principal platforms and their representative agents. Disease-specific evidence is presented in Section 5, and the key properties are listed in Table 1.

### 4.1. Oncolytic Adenovirus

Adenovirus serotype 5 is a nonenveloped, double-stranded DNA virus that replicates episomally without integrating into the host genome. It offers a large transgene capacity and has a well-characterized safety profile [14]. Two engineering strategies dominate. Conditionally replicating adenoviruses contain the E1 genes under a tumor-specific promoter, as in the telomerase-specific series developed by our group: OBP-301, the green fluorescent protein–expressing OBP-401 [15,21]. Alternatively, replication is restricted by gene deletion, as in DNX-2401 (Delta-24-RGD), which harbors an E1A-CR2 deletion along with an RGD fiber modification for CAR-independent entry [13]. Of these platforms, oncolytic adenoviruses, particularly DNX-2401, have generated mature clinical data in malignant glioma, which highlights their relevance to CNS and spinal tumors [13].

### 4.2. Oncolytic Herpes Simplex Virus

Herpes simplex virus type 1 (HSV-1) is a large, enveloped, double-stranded DNA virus whose sizable genome accommodates multiple transgenes. In addition, its natural neurotropism is advantageous for nervous system tumors, provided that neurovirulence is adequately attenuated [11,12]. Talimogene laherparepvec, which is an HSV-1 deleted in ICP34.5 and ICP47 and armed with granulocyte–macrophage colony-stimulating factor, was approved for advanced melanoma in 2015 [11]. In addition, the third-generation, triple-mutated HSV-1 teserpaturev (G47Δ) was approved in Japan in 2021 for malignant glioma. It represents the first oncolytic virus licensed for a primary brain tumor [12]. Therefore, approval of G47Δ establishes a notable regulatory precedent for oncolytic virotherapy in the CNS.

### 4.3. Reovirus

Reovirus is a nonenveloped, double-stranded RNA virus that is naturally oncolytic and non-pathogenic in humans. It requires no genetic engineering for tumor selectivity [7]. Its preferential replication in cells with activated RAS signaling underlies its tumor tropism, and the clinical agent pelareorep (reovirus serotype 3 Dearing) has been evaluated in multiple malignancies [7]. Notably, intravenously administered reovirus can reach intracranial tumors and stimulate an inflammatory, T-cell–infiltrated microenvironment, thus illustrating a systemically deliverable platform for CNS disease [39].

### 4.4. Other Platforms

Several additional platforms have reached CNS clinical testing and have expanded the therapeutic repertoire. A recombinant poliovirus–rhinovirus chimera, PVSRIPO, was rendered non-neurovirulent and targets the poliovirus receptor CD155, which is upregulated in glioma cells. It shows durable long-term survival in recurrent glioblastoma when administered by convection-enhanced delivery (CED) [40]. The rat protoparvovirus H-1PV (ParvOryx) crosses the blood–brain/tumor barrier and was proven safe with signs of immunogenic activity in recurrent glioblastoma [41]. Engineered measles virus derivatives, which enter cells through CD46, have been tested intratumorally and in the resection cavity in recurrent glioblastoma [42]. Other platforms, including oncolytic vaccinia virus, vesicular stomatitis virus, and Newcastle disease virus, offer complementary properties, such as large payloads, rapid lytic kinetics, and systemic deliverability [7]. Collectively, this range of CNS experience provides a strong translational foundation for extending virotherapy to spinal and spinal cord tumors.

## 5. Current Evidence of Virotherapy in Spinal Tumor Types

Table 2 summarizes the current evidence for oncolytic virotherapy in various spinal tumor types, which we consider by compartment below.

### 5.1. Virotherapy for Primary Vertebral Bone Tumors

To date, no oncolytic virus has been tested in a spinal or paraspinal primary bone tumor in a dedicated clinical study. Only histological evidence is available, which has primarily been obtained from tumors arising at appendicular or soft-tissue sites [19]. Nevertheless, the evidence is substantial. In osteosarcoma, the telomerase-specific oncolytic adenovirus OBP-301 exerts antitumor activity in preclinical bone and soft-tissue sarcoma models [21,43]. Other platforms, including herpes simplex virus, measles virus, and reovirus, have shown preclinical activity in osteosarcoma [20]. In Ewing sarcoma, vesicular stomatitis virus and the adenovirus XVir-N-31 show preclinical efficacy, the latter in combination with T-cell therapy [19]. For chordoma, which is a tumor exhibiting a strong predilection for the spine and sacrum, intratumoral administration of the TGF-β–trap, armed oncolytic adenovirus AdAPT-001, produced a partial response in an injected metastatic lesion in a first-in-man phase I study (BETA PRIME) [44]. According to a 2002–2023 review, 194 sarcoma patients received OVs in 19 trials; however, spinal primary tumors were scarcely represented [19].

### 5.2. *Virotherapy for* Metastatic Spinal Tumors

Metastatic spinal disease is the most common spinal tumor, but the least studied with respect to virotherapy. No dedicated clinical trials of OVs in spinal metastasis have been reported [45]. Preclinical studies have provided indirect support. For example, oncolytic adenoviruses armed with anti-angiogenic or immunomodulatory transgenes reduced skeletal and visceral metastatic burden in animal models [46]. Two features make spinal metastases a rational target. First, stereotactic body radiotherapy, which is now central to spinal metastasis management, can act synergistically with OVs to enhance viral replication and immunogenic cell death [45]. Second, image-guided percutaneous approaches, or the surgical corridor created during separation surgery, offer practical routes for intratumoral delivery. Because immunotherapy becomes more prevalent in metastatic disease, the capacity of OVs to convert a “cold” vertebral metastasis into an immune-reactive lesion represents a compelling opportunity [45].

### 5.3. Spinal Cord Gliomas and Intramedullary Tumors

Spinal cord astrocytomas and ependymomas share much of their biology with their intracranial counterparts; thus, the extensive glioma virotherapy experience is directly informative. Oncolytic adenovirus DNX-2401, herpes simplex virus G47Δ, the poliovirus chimera PVSRIPO, parvovirus H-1PV, and engineered measles virus have been evaluated in malignant glioma, and G47Δ has been approved for this indication [12,13]. The most anatomically relevant precedent is the treatment of diffuse intrinsic pontine glioma (DIPG) with DNX-2401. In a phase I study, 12 children received an intratumoral infusion of the virus through a catheter placed in the cerebellar peduncle, followed by radiotherapy, which resulted in an acceptable safety profile and encouraging survival [47]. Because DIPG occupies eloquent, unresectable brainstem tissue that is contiguous with the spinal cord, this study establishes the feasibility and tolerability of delivering a replicating virus into functionally critical neural parenchyma, which is the central concern for intramedullary application; however, direct evidence in spinal cord tumors remains at a preclinical stage.

Extrapolating from intracranial glioma to the spinal cord nevertheless requires caution, because the two compartments differ in ways that could affect both efficacy and safety. Cerebrospinal-fluid dynamics differ along the neuraxis, which may alter the residence time, clearance, and distribution of an instilled or intrathecally delivered virus. The spinal cord also has a lower resident immune-cell density and a distinct microglial and perivascular immune composition compared with the brain, which could modify both antiviral clearance and the antitumor immune response on which oncolytic activity depends. Its segmental vascular supply and the properties of the blood–spinal cord barrier differ from those of the cerebral circulation, affecting systemic delivery and the extent of barrier disruption. Finally, the mechanical constraints of the rigid, non-expandable spinal canal make virus-induced inflammation and edema more likely to cause neurological injury than in the intracranial compartment (Section 6.3). These biological and anatomical differences temper the direct transfer of intracranial efficacy and safety data to the spine and should be resolved through dedicated preclinical spinal models.

### 5.4. Intradural–Extramedullary and Extradural Tumors: Selected Refractory Entities

Most intradural–extramedullary tumors are benign and curable by resection; however, two aggressive entities within this and the extradural compartment warrant consideration as virotherapy candidates, and both are supported by preclinical data. Malignant peripheral nerve sheath tumors, which arise from spinal nerve roots and paraspinal nerves, respond to oncolytic herpes simplex virus in orthotopic models. A single intratumoral injection of G47Δ into sciatic nerve tumors inhibited growth and prolonged survival in immunodeficient and immunocompetent hosts, and arming the virus with interleukin-12 further improved its efficacy [48]. Adenovirus- and measles-based platforms have shown comparable preclinical results in nerve sheath tumors, although limited nectin-1 expression can constrain herpesviral entry, which is consistent with the receptor considerations discussed earlier [34,49]. For malignant (anaplastic) meningioma, a lethal, treatment-refractory subset that may adhere to the cord and preclude gross-total resection, oncolytic HSV induces cytotoxicity and antitumor immunity in preclinical models, and its activity is enhanced by histone deacetylase inhibition [50]. Together with the enrichment of telomerase reactivation in aggressive meningiomas discussed in Section 2.4, these results identify a narrow but rational niche: the recurrent or malignant intradural lesion, in which conventional local control has failed. Clinical evidence in the spine is not yet available, and these entities remain investigational targets.

## 6. Delivery and Safety in the Spinal Canal

### 6.1. Routes of Administration

**Figure 3:**
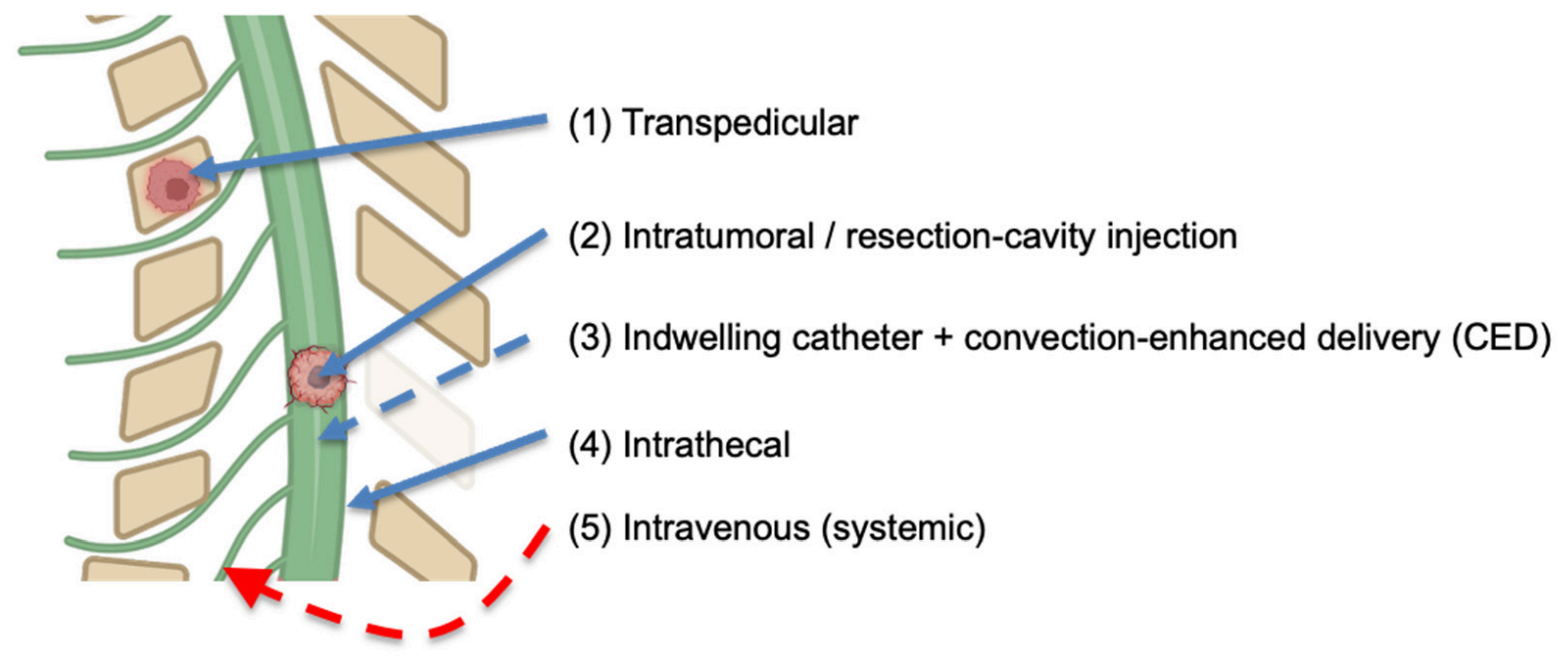
Delivery routes and safety considerations in the spinal canal. Candidate routes include transpedicular injection for vertebral tumors (1), intratumoral or resection-cavity injection (2), an indwelling catheter with convection-enhanced delivery (3), intrathecal administration (4), and intravenous delivery, which must cross the blood–spinal cord barrier (BSCB) (5). The rigid, nonexpandable canal potentially renders virus-induced inflammation or edema cord-compressing, and preexisting neutralizing antibodies favor repeatable local access.

The route of delivery must be matched to the tumor compartment (Figure 3), and it should allow repeated dosing, because the antitumor efficacy of oncolytic adenoviruses is enhanced by multiple treatments, rather than a single exposure [7]. For intramedullary tumors, two complementary strategies are envisaged: injection into the resection surface after maximal safe tumor removal and placement of an indwelling catheter at the time of surgery to enable subsequent percutaneous or reservoir-based re-dosing without repeated operation. For vertebral tumors, a transpedicular route provides reproducible, image-guided percutaneous access to the lesion. CED enables the distribution of virus through infiltrated cord parenchyma, while bypassing the blood–spinal cord barrier, as demonstrated by DNX-2401 administration for brainstem glioma [47]. Intrathecal administration can address cerebrospinal fluid-disseminated disease, but achieves limited parenchymal penetration [51], whereas intravenous delivery, although attractive for disseminated metastatic disease, must circumvent circulatory clearance, preexisting neutralizing antibodies, and the blood–spinal cord barrier [39,41].

### 6.2. Blood–Spinal Cord Barrier

The blood–spinal cord barrier is the spinal homolog of the blood–brain barrier and similarly restricts the entry of macromolecules and viral particles from the systemic circulation [51]. Tumor growth can disrupt this barrier focally, which produces the contrast enhancement observed upon imaging and permits some systemic access; however, this disruption is heterogeneous and incomplete. Therefore, an intact barrier remains a major obstacle to IV virotherapy [51]. Local routes (intratumoral injection and CED) circumvent the barrier altogether and currently offer the most reliable means of achieving therapeutic viral concentrations within spinal and spinal cord tumors [47,51].

### 6.3. Safety in the Confined Spinal Canal

The spinal canal imposes a safety consideration that is distinct from, and often more acute than that of the cranial compartment. Because the canal is rigid and nonexpandable, virus-induced inflammation, edema, or pseudoprogression may compress the cord and precipitate rapid, potentially irreversible neurological deterioration [47]. Therefore, intraspinal virotherapy requires conservative dosing, close neurological and imaging surveillance, and readiness for corticosteroid administration or surgical decompression. Platform choice also has safety implications. The natural neurotropism of herpes simplex virus must be adequately attenuated before intraspinal use, as discussed earlier. Antiviral immunity is double-edged, as preexisting neutralizing antibodies, which are highly prevalent against adenovirus serotype 5, limit systemic and repeated dosing, whereas repeated intratumoral injection of Delta-24-RGD is associated with reduced long-term benefit [52,53]. Nevertheless, the same immune activation underlies the systemic antitumor response, and local delivery keeps the neutralizing activity in the cerebrospinal fluid low, thereby preserving efficacy [52]. Because these agents are replication-competent, standard biosafety and viral-shedding precautions apply, although shedding has been considered minimal in CNS trials [47]. This tension between the need for repeated dosing and the antibody response it provokes requires delivery strategies that shield the virus during administration, which we discuss in the future directions (Section 8).

Adverse effects in the spine can be both local and systemic. Locally, viral replication and the ensuing inflammatory response could, in principle, cause intratumoral or peritumoral hemorrhage or necrosis extending into adjacent cord parenchyma, which within the confined canal might translate into motor or sensory deficits; the reported CNS (intracranial) experience has nonetheless been predominantly low-grade, with most events limited to transient, self-resolving neurological symptoms and manageable edema [47]. Systemically, oncolytic viruses commonly produce transient flu-like symptoms and cytokine release, and intravenous adenoviral delivery can cause dose-dependent hepatic enzyme elevation, so systemic administration carries a different toxicity profile from local injection [39,41].

### 6.4. Radiological and Histological Assessment of Oncolysis

Assessing the response of spinal and spinal cord tumors to virotherapy requires attention to features distinct from those of cytotoxic therapy. Histologically, effective oncolysis is marked by extensive tumor-cell necrosis and apoptosis, intranuclear and intracytoplasmic viral inclusion bodies, and a dense infiltrate of CD8+ T cells and other immune cells that reflects immunogenic cell death; some platforms produce characteristic cytopathic changes, such as the multinucleated syncytia induced by oncolytic measles virus [42]. On imaging, the same immune-mediated inflammation frequently produces transient tumor enlargement, increased edema, and new or increased contrast enhancement in the weeks after treatment—so-called pseudoprogression—which can mimic true tumor growth and must not be mistaken for treatment failure [13,47]. This distinction is critical in the spine, where inflammatory swelling within the rigid canal carries neurological risk (Section 6.3) and where size-based response criteria are unreliable. Serial MRI, ideally supplemented by perfusion or diffusion sequences and, where appropriate, metabolic imaging, together with close clinical correlation, is therefore required to distinguish productive oncolysis and inflammatory pseudoprogression from genuine progression; immunotherapy-adapted response criteria are better suited to this setting than conventional metrics.

## 7. Combination Strategies

### 7.1. Immune-Checkpoint Inhibitors

The combination of OVs with immune-checkpoint inhibitors is the most compelling. By lysing tumor cells and provoking immunogenic cell death, an oncolytic virus converts a “cold” tumor into a T-cell–inflamed lesion and upregulates PD-L1, thereby supplying the substrate on which checkpoint blockade acts [10]. Clinical data support this synergy. For example, DNX-2401 followed by pembrolizumab produced encouraging survival outcomes in recurrent glioblastoma [13], and OBP-301 combined with pembrolizumab yielded durable responses in esophagogastric adenocarcinoma, including in immunotherapy-refractory disease [54]. For the spine, this pairing is particularly relevant to metastases from checkpoint-responsive maladies, such as melanoma, renal cell carcinoma, and non-small-cell lung cancer, where local viral priming could sensitize an otherwise resistant vertebral lesion.

### 7.2. Radiotherapy and Chemotherapy

In this review, radiotherapy and chemotherapy are grouped as conventional cytotoxic adjuncts, although each augments virotherapy through a distinct mechanism. They are effective partners in the spine, where stereotactic body radiotherapy is already central to the management of metastatic disease. Ionizing radiation enhances viral uptake, replication, and intratumoral spread, and it amplifies immunogenic cell death; thus, the two modalities are mutually reinforcing [45]. This combination has been clinically established for a telomerase-specific platform. In a phase I study in esophageal cancer, repeated intratumoral OBP-301 with radiotherapy was well tolerated at doses ranging from 1 × 10^10^ to 1 × 10^12^ viral particles and produced tumor responses in the majority of evaluable patients [55]. Cytotoxic chemotherapy, via a separate mechanism, is likewise synergistic with OBP-301 [56]. This is a relevant consideration for chemotherapy-dependent histologies, such as osteosarcoma and Ewing sarcoma. In practice, intratumoral virotherapy may be integrated with SBRT and surgical corridors already used for spinal tumors.

### 7.3. Cellular Therapy

Chimeric antigen receptor (CAR) T-cell therapy has transformed hematologic malignancy outcomes, but has only achieved modest results in solid tumors, largely because of poor T-cell trafficking, limited tumor infiltration, and an immunosuppressive microenvironment [57]. OVs can address each of these barriers because they remodel the microenvironment, enhance T-cell infiltration, and can be armed with chemokines or cytokines to recruit and sustain adoptively transferred cells [57]. Unlike the strategies in Section 7.1 and Section 7.2, which have at least some spine-relevant clinical or preclinical support, this combination has no spine-specific or oncolytic-virus-in-spine preclinical grounding and rests entirely on general CAR-T and oncolytic-virus literature. We therefore present it strictly as a hypothetical future direction rather than an established combination strategy for spinal tumors.

## 8. Challenges and Future Perspectives

### 8.1. Toward Telomerase-Specific Adenoviral Virotherapy for the Spine

The telomerase-specific adenoviral platform is well matched to the demands of spinal neuro-oncology. Telomerase is reactivated in the predominant malignancies that involve the spine, including many sarcomas, metastatic carcinomas, and gliomas. Thus, hTERT-driven replication offers a rational, histology-agnostic route to tumor selectivity, with a notable exception for ALT-dependent tumors [15,16]. Because OBP-301 replicates in telomerase-positive tumor cells, while sparing the post-mitotic, telomerase-silent neurons and glia of the spinal cord, this platform is designed to precisely protect the tissue that must be preserved, which suggests a favorable therapeutic window for intramedullary use [15,21,43]. These properties make a coherent case for the preclinical evaluation of hTERT-driven oncolytic adenoviruses in spinal tumor models as a prelude to early-phase clinical study [21].

### 8.2. Enhancing Efficacy and Overcoming Delivery and Immunity Barriers

A central goal for spinal application is to widen the therapeutic window by simultaneously enhancing efficacy and limiting toxicity, while solving the practical problem that effective virotherapy often requires repeated dosing within a confined space. Efficacy can be increased by arming the virus with immunostimulatory transgenes—such as granulocyte–macrophage colony-stimulating factor or interleukin-12, the latter having improved outcomes in nerve sheath tumor models [11,48]—and by rational combination with immune-checkpoint inhibitors, radiotherapy, and chemotherapy, each of which augments viral activity through a complementary mechanism (Section 7). Repeated and convection-enhanced delivery further improves intratumoral spread and, for oncolytic adenovirus, is more effective than a single exposure [7,47]. Access can also be made durable and repeatable using the local delivery routes detailed in Section 6.1 (notably an indwelling catheter or reservoir with CED), which bypass the blood–spinal cord barrier [51]. Encapsulating the virus within extracellular vesicles is a further emerging strategy for systemic, repeatable delivery, because extracellular vesicle–cloaked oncolytic adenovirus evades neutralizing antibodies and, by bypassing CAR-dependent entry, can transduce CAR-low tumors following intravenous administration [58,59,60]; in parallel, nanomedicine-based carriers are being actively developed to improve drug delivery across the blood–brain and blood–spinal cord barriers in primary and secondary brain tumors and may offer complementary vehicles for delivering oncolytic agents or combination payloads to the CNS [61]. Toxicity, in turn, is reduced chiefly by tighter targeting: transcriptional control by the hTERT promoter and the Δ24 deletion confine replication to tumor cells, adequate attenuation limits the neurovirulence of herpesviral platforms, and local rather than systemic delivery minimizes off-target exposure while keeping neutralizing-antibody activity in the cerebrospinal fluid low [12,13,15,52]. Combined with biomarker-guided patient selection (Section 8.3), these measures could, in principle, extend telomerase-specific oncolytic adenoviruses such as OBP-301 to disseminated spinal metastatic disease, although extracellular-vesicle delivery remains at a preclinical stage, and the low yield of current extracellular-vesicle production and purification methods limits its near-term clinical application [60].

### 8.3. Biomarkers and Patient Selection

The heterogeneity of viral susceptibility requires biomarker-guided patient selection rather than empirical treatment. For adenovirus-based agents, tumor expression of the coxsackievirus–adenovirus receptor and, particularly, telomerase or hTERT, can identify the tumors most likely to respond, whereas the alternative-lengthening-of-telomeres phenotype, which is common in osteosarcoma and in a subset of astrocytomas, can predict resistance to hTERT-dependent viruses [31,35,36]. Incorporating these assays into eligibility criteria can identify patient populations for likely responders and spare others ineffective and potentially hazardous treatments. Fluorescence-based assessment of viral susceptibility with OBP-401 offers a practical route for stratification [18].

### 8.4. Limitations and Safety Considerations

Several biological features of the platform warrant explicit consideration as potential limitations. First, telomerase is not entirely tumor-restricted: it remains active in proliferating stem and progenitor cells, including hematopoietic, germline, and neural stem/progenitor populations. In the adult spinal cord, telomerase-active cells are largely confined to the ependymal/central-canal niche, whereas the surrounding neurons and mature glia are post-mitotic and telomerase-silent. hTERT-promoter activity—and hence viral replication—is nonetheless substantially lower in normal stem cells than in tumor cells, and first-in-human trials of OBP-301 across solid tumors reported a favorable safety profile without stem-cell-related dose-limiting toxicity [16]. On-target, off-tumor replication in telomerase-active normal cells therefore remains a theoretical risk that requires vigilance, particularly for local delivery adjacent to the central canal. Second, the adenoviral E1A protein carries intrinsic risks: through its CR2 domain it binds retinoblastoma protein and drives quiescent cells into S phase, it can cooperate with cellular oncogenes, and it antagonizes interferon-mediated antiviral responses. In OBP-301, E1A is transcriptionally restricted by the hTERT promoter, confining these activities to telomerase-positive tumor cells; the alternative Δ24 (E1A-CR2–deleted) design used in DNX-2401 restricts replication to cells with a defective retinoblastoma pathway [13]. Because these viruses are non-integrating, insertional mutagenesis is unlikely, but low-level E1A expression and interferon antagonism in non-target cells remain safety considerations that justify monitoring. Third, immune responses represent an intrinsic constraint: virus-neutralizing antibodies—highly prevalent against adenovirus serotype 5—limit systemic and repeated dosing but can be mitigated by local delivery, serotype switching, or extracellular-vesicle encapsulation, while the same immune activation also contributes to the therapeutic antitumor response. The procedural hazards of virus-induced inflammation within the rigid spinal canal are addressed in Section 6.3.

### 8.5. Barriers to Launching Spinal Virotherapy Trials

A candid assessment must acknowledge that no oncolytic virus has yet entered a clinical trial dedicated to a primary or metastatic spinal tumor; the supporting evidence is extrapolated almost entirely from intracranial gliomas and from sarcomas arising at appendicular or soft-tissue sites (Section 5 and Section 8.1). Several structural barriers explain this gap and temper expectations of near-term clinical application. First, spinal and spinal cord tumors are individually rare. Primary vertebral malignancies and intramedullary tumors each account for only a small fraction of neoplasms, so accrual is slow and adequately powered randomized trials are difficult to mount. Second, these tumors are histologically diverse—spanning osteosarcoma, Ewing sarcoma, chordoma, varied metastatic carcinomas, astrocytoma, ependymoma, and nerve sheath tumors—and each histology differs in receptor expression, telomere-maintenance mechanism, and viral susceptibility. This diversity fragments an already small population and complicates uniform eligibility criteria and dosing. Third, the eloquent location requires that efficacy be judged against the preservation of neurological function. Virus-induced inflammation, edema, or pseudoprogression within the rigid spinal canal can precipitate rapid neurological deterioration and is difficult to distinguish from true progression on imaging, so trials require standardized neurological endpoints, serial functional assessment, and predefined imaging criteria that are not yet established for the spine. Finally, replication-competent agents entail complex good-manufacturing-practice production and biosafety requirements that add cost and regulatory burden. Together, these barriers indicate that clinical translation will be incremental rather than immediate and must be preceded by dedicated preclinical studies in spinal tumor models.

### 8.6. Clinical Translation and Trial Design

The treatment of spinal tumors with virotherapy faces structural difficulty, as these tumors are rare and histologically diverse, which hinders the design of conventional randomized trials. Pragmatic solutions include histology-agnostic basket designs based on a shared biomarker, such as telomerase positivity, multicenter collaboration, and the use of co-primary endpoints that assess tumor control and neurological function preservation, which is the outcome that matters most in the spine. Because of the acute consequences of cord compromise, early-phase studies will require conservative dose escalation, rigorous neurological and imaging surveillance, and predefined criteria for corticosteroid use or surgical decompression. Careful attention to these design features is necessary to evaluate efficacy without exposing patients to undue neurological risk.

## 9. Conclusions

Spinal and spinal cord tumors, which span primary and metastatic vertebral tumors, intramedullary gliomas, and selected refractory intradural lesions, are difficult to treat because the surrounding spinal cord and nerve roots are affected by aggressive resection and high-dose irradiation. Oncolytic virotherapy represents a rational approach because it lyses tumor cells selectively, elicits systemic antitumor immunity, and spares normal neural tissue; however, clinical evidence in the spine is lacking. The current rationale is based on evidence at the histological level from sarcomas and other tumor types, on extensive experience with glioma, including the anatomically proximate treatment of DIPG, and on preclinical activity in nerve sheath and meningioma models. Of the available platforms, the telomerase-specific oncolytic adenovirus series, OBP-301 and its derivatives, is well suited to the spine because replication driven by the hTERT promoter confers selectivity independent of histology, while sparing the spinal cord, which lacks telomerase activity. Realizing this potential will require solutions tailored to the spinal canal, such as repeatable delivery through indwelling catheters or CED, encapsulation in extracellular vesicles to overcome antiviral immunity, patient selection guided by biomarkers, and trial designs that safeguard neurological function. By carefully considering these factors, and pending dedicated preclinical and early-phase evaluation, virotherapy offers a promising avenue for a group of tumors whose current treatment options are limited.

More broadly, the telomerase-specific, histology-agnostic design is not spine-specific. Telomerase is reactivated in the large majority of human cancers, and OBP-301 has been evaluated across diverse solid tumors, including esophageal, gastric, and other malignancies [16,54,55]. The spine represents a particularly compelling niche because of its acute need for tumor-selective, neural-sparing local therapy, but the principles reviewed here—selective oncolysis, immunogenic cell death, biomarker-guided selection, and rational combinations—apply broadly across solid-tumor oncology; conversely, experience gained in the anatomically constrained spine may inform virotherapy for other eloquent or immunologically cold tumors.

## Figures and Tables

**Table 1:** Principal oncolytic virus platforms relevant to spinal and spinal cord tumors.

Platform	Genome	Transgene Capacity	Primary Selectivity Mechanism	Representative Agent(s)	CNS Status
Adenovirus (Ad5)	dsDNA	Large	*hTERT* promoter; *E1A*-CR2 (Δ24) deletion; entry via CAR (RGD for CAR independence)	OBP-301, OBP-401; DNX-2401	Mature glioma clinical data (DNX-2401)
Herpes simplex virus-1	dsDNA	Very large	Attenuation (ICP34.5/ICP47/ICP6): entry via nectin-1/HVEM	Talimogene laherparepvec; teserpaturev (G47Δ)	Approved for malignant glioma in Japan (G47Δ)
Reovirus	dsRNA	None (unmodified)	RAS-pathway–activated cells	Pelareorep	Systemic delivery reaches intracranial tumors
Poliovirus–rhinovirus chimera	ssRNA(+)	Limited	Non-neurovirulent; CD155 (upregulated on glioma)	PVSRIPO	Phase I/II in recurrent glioblastoma
Parvovirus H-1	ssDNA	Limited	Oncotropic; crosses the blood–brain/tumor barrier	ParvOryx (H-1PV)	Phase I/IIa in recurrent glioblastoma
Measles virus	ssRNA(−)	Moderate	Entry via CD46 (overexpressed on tumors)	MV-CEA, MV-NIS	Phase I in recurrent glioblastoma

**Table 2:** Current evidence for oncolytic virotherapy in various spinal tumor types.

Compartment	Representative Histology	Oncolytic Platform(s)	Evidence Level	Ref.
Primary vertebral bone	Osteosarcoma	Telomerase-specific adenovirus (OBP-301); HSV; measles; reovirus	Preclinical (histology-level; non-spinal models)	[20,21,43]
Primary vertebral bone	Ewing sarcoma	Vesicular stomatitis virus (VSVΔM51); adenovirus (XVir-N-31)	Preclinical (histology-level; non-spinal models)	[19]
Primary vertebral bone	Chordoma	TGF-β–trap adenovirus (AdAPT-001)	Clinical (phase I; PR in an injected metastatic lesion)	[44]
Metastatic spinal	Carcinoma metastases	Armed oncolytic adenovirus (decorin)	Preclinical (bone-metastasis models); no spine-specific trials	[45,46]
Spinal cord glioma/IMSCT	Astrocytoma, ependymoma	DNX-2401; G47Δ; PVSRIPO; H-1PV; measles (intracranial/DIPG)	Clinical for intracranial glioma; extrapolated to the cord	[12,13,40,41,42,47]
Intradural–extramedullary	MPNST	Oncolytic HSV (G47Δ)	Preclinical (orthotopic)	[48,49]
Intradural–extramedullary	Malignant meningioma	Oncolytic HSV + HDAC inhibition	Preclinical	[50]

For primary vertebral bone tumors, no oncolytic virus has been administered to a vertebral (spinal) lesion; the evidence is histological, derived from the same tumor types studied predominantly at appendicular or soft-tissue sites (osteosarcoma, Ewing sarcoma) or, for chordoma, from an accessible metastasis in an early-phase trial. IMSCT, intramedullary spinal cord tumor; DIPG, diffuse intrinsic pontine glioma; MPNST, malignant peripheral nerve sheath tumor; PR, partial response; HDAC, histone deacetylase.

## Data Availability

No new data were created or analyzed in this study. Data sharing is not applicable to this article.

## Abbreviations

The following abbreviations are used in this manuscript:

ALT	alternative lengthening of telomeres
BSCB	blood–spinal cord barrier
CAR	coxsackievirus–adenovirus receptor
CED	convection-enhanced delivery
CNS	central nervous system
DIPG	diffuse intrinsic pontine glioma
GFP	green fluorescent protein
HDAC	histone deacetylase
HSV	herpes simplex virus
hTERT	human telomerase reverse transcriptase
HVEM	herpesvirus entry mediator
IMSCT	intramedullary spinal cord tumor
MPNST	malignant peripheral nerve sheath tumor
NOMS	neurologic, oncologic, mechanical, and systemic
OV	oncolytic virus
PD-L1	programmed death-ligand 1
PR	partial response
RGD	arginine–glycine–aspartate (motif)
SBRT	stereotactic body radiotherapy
SINS	Spinal Instability Neoplastic Score
TGF-β	transforming growth factor-β
VSV	vesicular stomatitis virus
WBB	Weinstein–Boriani–Biagini (staging system)
WHO	World Health Organization
